# Angina e Ensaio ORBITA 2: Reflexões sobre o Futuro do Tratamento da Angina

**DOI:** 10.36660/abc.20230848

**Published:** 2024-07-15

**Authors:** Eduardo Bello Martins, Eduardo Gomes Lima, Jaime Paula Pessoa Linhares, Henrique Trombini Pinesi, Fabio Grunspun Pitta, Carlos Vicente Serrano

**Affiliations:** 1 Universidade de São Paulo Faculdade de Medicina Instituto do Coração São Paulo SP Brasil Instituto do Coração (InCor) – Hospital das Clínicas HCFMUSP – Faculdade de Medicina – Universidade de São Paulo, São Paulo, SP – Brasil; 2 Hospital Israelita Albert Einstein Sao Paulo SP Brasil Hospital Israelita Albert Einstein, Sao Paulo, SP – Brasil; 3 Hospital OTO Aldeota Kora Saúde Fortaleza CE Brasil Hospital OTO Aldeota – Kora Saúde, Fortaleza, CE – Brasil

**Keywords:** Coronariopatia, Angina Estável, Revascularização Miocárdica

O objetivo do tratamento da doença arterial coronariana crônica (DAC) é melhorar o prognóstico e a qualidade de vida do paciente. Nesse contexto, o tratamento clínico otimizado (em inglês, *optimal medical therapy* , OMT) é fundamental, e a revascularização realizada principalmente em pacientes cujos sintomas não são controlados pela OMT. A intervenção coronária percutânea (ICP) tem sido frequentemente realizada nos últimos 40 anos, mas os dados disponíveis não corroboram sua indicação com o objetivo de melhorar o prognóstico. Além disso, dados sobre a ICP e melhora de sintomas são controversos. Contudo, as diretrizes internacionais mais recentes sobre a DAC ainda defendem a utilização da ICP em pacientes com sintomas refratários à OMT.^1^

Os ensaios mais contemporâneos na DAC avaliaram, cuidadosamente, o efeito da ICP sobre a angina. O estudo MASS ( *Medicine, Angioplasty and Surgeon Study* ) II mostrou uma alta taxa de pacientes sem sintomas de angina com revascularização *versus* OMT em um ano.^2^ Contudo, outros dados põem em questão a eficácia da ICP nos sintomas. O ensaio COURAGE ( *Clinical Outcomes Using Revascularization and Aggressive Drug Evaluation* ) e o ensaio FAME 2 revelaram um alívio inicial dos sintomas logo após a ICP, mas a diferença em relação à OMT não se manteve após três a cinco anos de seguimento, respectivamente.^3 - 5^ Uma subanálise do ensaio BARI-2D avaliou o estado de saúde e os sintomas dos pacientes randomizados à revascularização ou a tratamento com medicamento. No BARI-2D, os pacientes tratados com revascularização cirúrgica mostraram melhora dos sintomas, o que não ocorreu entre os submetidos à angioplastia em um acompanhamento em longo prazo. Outro estudo conduzido por Hambrecht et al.^6^ comparou a ICP à reabilitação física, que também é um componente chave na OMT, e mostrou uma melhor tolerância ao exercício no grupo tratado exclusivamente com reabilitação.^6^

Mais recentemente, o ensaio ISCHEMIA ( *International Study of Comparative Health Effectiveness with Medical and Invasive Approaches* ) demonstrou, em pacientes com isquemia grave detectada por métodos não invasivos, uma melhora na qualidade de vida com a revascularização (ICP ou *bypass* da artéria coronária).^7^ O melhor desempenho da estratégia invasiva ocorreu em pacientes com sintomas mais frequentes e mais limitantes, analisados no estudo pelo escore SAQ ( *Seattle Angina Questionnaire* ).

## Ensaio ORBITA

Embora os estudos mencionados anteriormente tenham demonstrado alguma melhora na angina após a revascularização do miocárdio, uma das suas principais limitações é por serem estudos abertos, com possível efeito placebo. Nesse aspecto, o estudo ORBITA mostrou que não houve benefício da ICP em comparação a um procedimento placebo em relação ao desfecho primário de um aumento no tempo de exercício.^8^ O ORBITA foi um estudo muito bem conduzido que demonstrou a possibilidade de um componente placebo e falta de uma melhora de sintomas pela ICP. No entanto, várias limitações já discutidas na literatura podem ter influenciado esses resultados, tais como o fato de o estudo haver incluído pacientes com sintomas não limitantes, ausência de isquemia em quase um quarto dos participantes, e o curto período de acompanhamento.

Em 2023, o estudo ORBITA 2 foi finalmente publicado no NEJM e abordou, pela primeira vez, a ICP como uma monoterapia para alívio da angina em um ensaio controlado com placebo.^9^ O estudo fornece mais uma peça do quebra-cabeça do controle da angina e supera as limitações do ORBITA. O ORBITA 2 foi um estudo multicêntrico, duplo-cego, randomizado, controlado por placebo, de iniciativa do investigador, conduzido em 14 locais no Reino Unido e incluiu 301 participantes. Os pacientes incluídos apresentavam angina ou sintomas equivalentes, evidência anatômica de estenose coronária grave, e evidência objetiva de isquemia com base em exames de imagens não invasivos ou análise invasiva da fisiologia coronariana. Primeiramente, medicamentos antianginosos foram descontinuados (o número mediano de drogas antianginosas foi de apenas 1) e os participantes foram orientados a usar um aplicativo de smartphone exclusivo para relatar a presença ou a ausência de angina e o número de episódios de angina diariamente. Os pacientes também preencheram questionários validados sobre sintomas e qualidade de vida. A classificação funcional da Sociedade Canadense de Cardiologia (CCS, *the Canadian Cardiovascular Society* ) foi avaliada, e realizados teste ergométrico e ecocardiografia de estresse com dobutamina. Em seguida, os pacientes entraram em um período de duas semanas de pré-randomização, durante o qual relataram o número de episódios de angina diariamente, pelo aplicativo do smartphone. Os pacientes eram elegíveis a prosseguir com a randomização se relatassem pelo menos um episódio de angina durante a fase de avaliação de sintoma.

Subsequentemente, angiografia coronariana foi realizada enquanto os pacientes escutavam música por fones de ouvido, e os pacientes elegíveis receberam sedação leve. Na fase pré-randomização, foram realizadas avaliações fisiológicas invasivas em cada vaso com uma estenose de pelo menos 50% do diâmetro, baseada na estimativa visual. Somente os pacientes com pelo menos uma lesão isquêmica foram considerados elegíveis para inclusão e randomização para ICP ou procedimento placebo. O desfecho primário foi um escore de angina, calculado com o número de episódios de angina relatado pelo paciente em um dia e o número de medicamentos antianginosos prescritos para o paciente naquele dia.

Após um seguimento cego de 12 semanas, os resultados do ORBITA 2 mostraram um benefício quanto aos sintomas com o ICP. O escore médio de angina em 12 semanas foi 2,9 no grupo ICP e 5,6 no grupo placebo (p<0,001). Contudo, o tamanho do efeito de alívio da angina foi modesto, uma vez que a frequência diária de angina foi somente 0,4 menos na ICP (0,3 episódios no grupo ICP e 0,7 no grupo placebo) e 59% dos pacientes ainda apresentavam sintomas residuais após a ICP. O aumento no tempo de exercício na esteira foi de somente 59,5 segundos e similar ao que se alcança com um único medicamento antianginoso, conforme destacado pelos autores. Outra limitação é o curto período de acompanhamento, uma vez que resultados em longo prazo da ICP para angina podem mudar devido à re-estenose, neoaterosclerose ou reações inflamatórias, e disfunção endotelial induzida por *stent* . Vale ressaltar que o benefício foi observado logo após a ICP e sustentado durante o período do estudo. Ainda, 40 pacientes no grupo ICP não apresentavam angina em comparação a somente 15 pacientes no grupo OMT, o que ressalta a importância de se identificar preditores de melhora de sintomas após uma ICP, de modo que os médicos possam oferecer uma revascularização mais personalizada.

O uso do smartphone para monitoramento clínico e de sintomas no estudo ORBITA-2 merece atenção. Intervenções Digitais de Saúde (IDSs) estão cada vez mais sendo incorporadas à prática clínica. Uma IDS permite que o médico avalie melhora a carga do sintoma pelo simples registro do número de episódios de angina por dia, sua associação e seu impacto sobre o exercício, melhora com uso de medicamento e angina limitante.

## Conclusões

Em geral, o conjunto de evidências valida um papel da ICP no alívio de sintomas. No entanto, é preciso cautela antes de se apressar à realização de revascularização. A identificação de sintomas e de angina é um dilema diagnóstico. Em alguns pacientes, a dor torácica, causada por condições musculoesqueléticas ou dispépticas, pode ser mal interpretada como uma resposta isquêmica em um teste invasivo ou não invasivo. Outro ponto crucial é que o fluxo coronariano é regulado pela microcirculação, reconhecida como uma causa de sintomas e pior prognóstico na DAC.^10^ Infelizmente, uma avaliação abrangente da fisiologia da artéria coronária é raramente realizada na prática clínica, a qual poderia ter elucidado a causa dos sintomas residuais em 59% dos pacientes após a ICP no ORBITA 2. De fato, a avaliação da microcirculação e vasoespasmo melhorou sintomas, conforme mostrado no ensaio CorMicA.^11^ Ainda, vale mencionar que um componente placebo da revascularização miocárdica é, até certo ponto, responsável pela melhora de sintomas, já que o tamanho do efeito foi menor que os resultados observados em ensaios não cegos.

**Figura 1:**
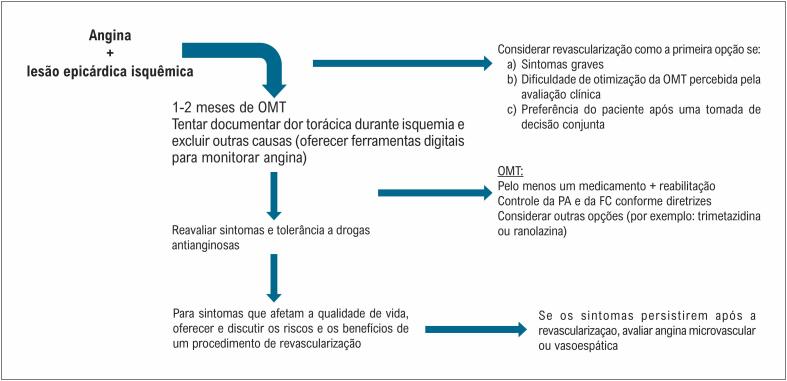
Algoritmo de tratamento da angina; OMT: Optimal medical therapy; PA: pressão arterial; FC: frequência cardíaca.

Após uma cuidadosa avaliação, acreditamos que os estudos ORBITA poderiam mudar as recomendações das diretrizes atuais em se realizar uma ICP somente a pacientes com sintomas refratários apesar da OMT, e propomos um algoritmo de manejo da angina ( Figura 1 ). No entanto, a OMT deveria continuar como a primeira opção na maioria dos casos, por ser segura, conseguir aliviar sintomas de maneira tão eficaz como a ICP, permite que os cardiologistas avaliem melhor os sintomas relatados e discutam as opções disponíveis (desde a reabilitação física até o tratamento medicamentoso). A ICP, ou mesmo o *bypass* da artéria coronária, são opções para pacientes com lesões isquêmicas epicárdicas (usando índices hemodinâmicos específicos para lesão epicárdica) e sintomas persistentes de angina apesar da OMT ou para casos em que a intolerância a medicamentos antianginosos for identificada ou altamente esperada. Alguns pacientes, após uma tomada de decisão em conjunto, podem não querer tomar medicamentos adicionais, e expressarem uma preferência pela revascularização como a primeira opção após considerações relativas à complexidade e aos riscos do procedimento. Finalmente, o estudo ORBITA 2 destaca um importante papel das ferramentas digitais modernas no melhor monitoramento da angina, o que deveria ser mais bem explorado em estudos clínicos futuros.

Fonte: primeiro autor.
