# Supplementary material for: Angina e Ensaio ORBITA 2: Reflexões sobre o Futuro do Tratamento da Angina
Source: Arq Bras Cardiol. 2024 Jul 15;121(6):e20230848. [Article in Portuguese] doi: 10.36660/abc.20230848 (PMC12092044; doi:10.36660/abc.20230848)
Supplement: Translation

# Translation: Angina Pectoris and Orbita 2 Trial: Reflections on the Future of Angina Treatment

**DOI:** 10.36660/abc.20230848i

**Authors:** Eduardo Bello Martins, Eduardo Gomes Lima, Jaime Paula Pessoa Linhares, Henrique Trombini Pinesi, Fabio Grunspun Pitta, Carlos Vicente Serrano

**Affiliations:** 1 São Paulo SP Brazil Instituto do Coração (InCor) – Hospital das Clínicas HCFMUSP – Faculdade de Medicina – Universidade de São Paulo, São Paulo, SP – Brazil; 2 Sao Paulo SP Brazil Hospital Israelita Albert Einstein, Sao Paulo, SP – Brazil; 3 Fortaleza CE Brazil Hospital OTO Aldeota – Kora Saúde, Fortaleza, CE – Brazil

**Keywords:** Coronary Disease, Angina Pectoris, Myocardial Revascularization

Treatment goal of chronic coronary disease (CCD) is to improve patient prognosis and quality-of-life. On this matter, optimal medical therapy (OMT) is paramount and revascularization is performed mostly for patients in which symptoms are not controlled by OMT. Although percutaneous coronary intervention (PCI) has been frequently performed in the last 40 years, available data do not support its indication for prognosis and data on PCI and symptom improvement are controversial. Nevertheless, the more recent international guideline on CCD still supports PCI in patients with symptoms refractory to OMT.^1^

The most contemporary trials in CCD carefully evaluated the effect of PCI on angina. The MASS (Medicine, Angioplasty and Surgery Study) II trial showed a higher rate of patients free of anginal symptoms with revascularization versus OMT at one year.^2^However, other data put into question the long-term efficacy of PCI on symptoms. The Clinical Outcomes Using Revascularization and Aggressive Drug Evaluation (COURAGE) and FAME 2 trials revealed initial symptom relief soon after PCI, but the difference in relation to OMT was not sustained after three and five years of follow-up, respectively.^3 - 5^A subanalysis of the BARI-2D trial evaluated the health status and symptoms of patients randomized to revascularization or drug treatment. In BARI-2D, patients treated with surgical revascularization showed improvement of symptoms, which did not occur among those who underwent angioplasty in a long-term follow-up. Another study conducted by Hambrecht et al.^6^compared PCI to physical rehabilitation, which is also a core component of OMT, and showed better tolerance to exercise in the group treated with physical rehabilitation alone.^6^

More recently, the ISCHEMIA trial (International Study of Comparative Health Effectiveness with Medical and Invasive Approaches) demonstrated, in patients with severe ischemia in non-invasive methods, a quality-of-life improvement with revascularization (either with PCI or coronary artery bypass grafting).^7^The best performance of the invasive strategy occurred in patients with more frequent and limiting symptoms, analyzed in the study by the Seattle Angina Questionnaire (SAQ) Summary Score.

## The ORBITA trial

Although the above-mentioned studies have demonstrated some improvement in angina after myocardial revascularization, one of their major limitations is the open nature and a possible placebo effect. In this regard, the ORBITA study showed no benefit of PCI versus a placebo procedure on the primary end-point of an increase in exercise time.^8^ORBITA was a well conducted study that demonstrated the possibility of a placebo component and lack of symptom improvement by PCI. However, several limitations could have influenced these results and have been discussed in the literature, such as the fact that it included patients with non-limiting symptoms, no ischemia in almost a quarter of the participants and short follow-up.

In 2023, the ORBITA 2 trial was finally published in NEJM and addressed for the first time the PCI as a monotherapy for angina relief at a placebo-controlled trial.^9^It provides another piece of the puzzle of angina control and overcomes some of the first ORBITA limitations. ORBITA 2 was an investigator-initiated, multicenter, double-blind, randomized, placebo-controlled trial that was conducted at 14 sites in the United Kingdom and included 301 participants. Patients enrolled had angina or equivalent symptoms, anatomic evidence of severe coronary stenosis and objective evidence of ischemia *based on noninvasive imaging or invasive coronary physiology testing.* First, antianginal medications were discontinued (median number of antianginal drugs was only 1) and the participants were instructed to use a dedicated smartphone application to report the presence or absence of angina and the number of angina episodes daily. *Patients also completed validated questionnaires on symptoms and quality of life. The Canadian Cardiovascular Society (CCS) functional classification was assessed, treadmill exercise test and dobutamine stress echocardiography were also performed. The patients then entered a two-week pre-randomization period during which they reported the number of episodes of angina daily, through the smartphone application. Patients were eligible to proceed to randomization if they reported at least one episode of angina during the symptom assessment phase.*

Subsequently, coronary angiography was performed while patients listened to music through headphones, and eligible patients received mild sedation. In the pre-randomization phase, invasive physiological assessments were performed in each vessel with a stenosis of at least a 50% diameter based on visual estimation. Only those with at least one ischemic lesion were deemed eligible for inclusion and randomization for PCI or a placebo-procedure. The primary outcome was an angina score, calculated with the number of angina episodes reported by the patient on a given day and the number of antianginal medications prescribed for the patient on that day.

After a 12-week blinded follow-up the results of ORBITA 2 showed a symptomatic benefit with PCI. Mean angina score at 12 weeks was 2.9 in the PCI group and 5.6 in the placebo group (P<0.001). Still, the effect size of angina relief was modest since the daily frequency of angina was only 0.4 less in PCI (0.3 episodes in the PCI group and 0.7 in the placebo group) and 59% of patients still had residual symptoms after PCI. The increase in treadmill exercise time was only 59.5 seconds and similar to what is achieved with a single antianginal medication as highlighted by the authors. Another limitation is the short follow-up as long-term results of PCI for angina may change due to restenosis, neoatherosclerosis or inflammatory reactions and endothelial dysfunction induced by the stent. Of note, the benefit was seen soon after PCI and sustained during the study follow-up. Also, 40 patients in the PCI group were free from angina compared to only 15 patients in the OMT group which underscores the importance of identifying predictors of symptom improvement after a PCI, so that clinicians can offer a more personalized revascularization.

*The use of smartphone for clinical monitoring and symptom surveillance in ORBITA-2 deserves attention. Digital health interventions (DHIs) are increasingly being incorporated into daily practice. A DHI allows the clinician to better assess symptom burden by easily documenting the number of angina episodes per day, its association and impact on exercise time, improvement with medication use and limiting angina.*

## Conclusions

Overall, the body of evidence validates a role for PCI in symptomatic relief. Nevertheless, caution is needed before rushing into a revascularization procedure. The identification of symptoms and angina pectoris is a diagnostic dilemma. In some patients, chest pain caused by musculoskeletal or dyspeptic conditions, may be misinterpreted as an ischemic response on an invasive or non-invasive test. Another crucial point is that coronary flow is regulated by microcirculation, which is recognized as a cause of symptoms and worse prognosis in CCD.^10^Unfortunately, a comprehensive assessment of coronary artery physiology is rarely performed in clinical practice, which could have elucidated the cause of the residual symptoms in 59% of patients after PCI in ORBITA 2.

In fact, assessment of microcirculation and vasospasm improved symptoms as showed in the CorMicA trial.^11^Also, it is worth mentioning that a placebo component of myocardial revascularization is, to a certain extent, responsible for symptom improvement since the effect size was smaller than the results observed in unblinded trials.

After careful consideration, we believe the ORBITA studies could change current guideline recommendations of offering PCI only for patients with refractory symptoms despite OMT and propose an algorithm for angina management ( Figure 1 ). However, OMT should remain the first option in most cases since it is safe, can relieve symptoms as effectively as PCI, and allows the cardiologist to better evaluate the reported symptoms and discuss all available options (from physical rehabilitation to drug treatment). PCI, or even CABG, are options for those with epicardial ischemic lesions (using hemodynamic indexes specific for epicardial lesion) and persistent anginal symptoms despite OMT, or in cases where intolerance to antianginal medications is identified or highly expected. Some patients, after a shared decision-making process, may be unwilling to take additional pills and express a preference for revascularization as the first option after considerations relative to procedure complexity and risks. Lastly *, the ORBITA 2 trial highlights a major role of modern digital tools in better monitoring angina, which should be further explored in future trials.*

**Figure 1:** Angina treatment algorithm. OMT: optimal medical therapy; BP: blood pressure; HR: heart rate.

Source: first author.
